# Repeatable and deterministic all electrical switching in a mixed phase artificial multiferroic

**DOI:** 10.1038/s41598-022-09417-0

**Published:** 2022-03-29

**Authors:** W. Griggs, T. Thomson

**Affiliations:** NEST Research Group, The Department of Computer Science, The University of Manchester, Oxford Road, Manchester, M13 9PL UK

**Keywords:** Ferroelectrics and multiferroics, Magnetic properties and materials, Phase transitions and critical phenomena

## Abstract

We demonstrate a repeatable all-electric magnetic switching behaviour in a PMN-PT/FeRh thin film artificial multiferroic. The magnitude of the effect is significantly smaller than expected from conventional thermomagnetic switching of FeRh thin films and we explore properties of the PMN-PT/FeRh system in order to understand the origin of this reduction. The data demonstrate the importance of the crystallographic phase of PMN-PT and show how a phase transition at ~ 100 °C modifies the magneto-electric coupling. We demonstrate a large strain remanence effect in the PMN-PT substrate, which limits the magnetoelectric coupling on successive cycling of the applied electric field.

## Introduction

Multiferroic systems with large magnetoelectric coupling constants offer exciting potential for implementation in next-generation devices for data storage^1,2^, sensing^3,4^, and neuromorphic computing^5,6^. The promise of these systems resides in their ability to generate large and bistable magnetic responses to applied voltages, which are simple to implement and thus provide a mechanism for switching with enhanced efficiency. Some progress to identify and understand single phase magnetoelectric multiferroics was made during the during the 1960s and 1970s^7–9^, but this progress decelerated thereafter, in part a consequence of their sparsity in nature; the simultaneous preference for vacant d-orbitals for ferroelectricity and partially filled d-orbitals for ferromagnetism specifies a soft mutual exclusivity between the two phenomena^10^. However, recent advances in atomic engineering at the interfaces between heterogeneous thin films have driven a renewed interest in magnetoelectric multiferroics, albeit with the proviso that the systems now studied are typically multi-layered structures which derive their multiferroic properties through interfacial coupling^11–13^. These artificial multiferroics provide much improved performance compared to their single-phase counterparts, usually producing magnetoelectric coupling constants which are larger by more than one order of magnitude^14^.

One such system comprises an FeRh thin film on a piezoelectric substrate^15–27^. Equiatomic FeRh undergoes a first-order metamagnetic phase transition (MPT) from G-type antiferromagnetic (AF) ordering to ferromagnetic (FM) ordering at ∼ 100 °C^28^, and the MPT coincides with a ∼ 0.3% lattice constant increase in 3 dimensions leading to ∼ 1% volumetric expansion of the unit cell^29^. Thus, the coexistence of magnetic phases can be manipulated via clamping FeRh to a piezoelectric substrate, which behaves as a mechanical actuator. Frequently, [Pb(Mg_1/3_Nb_2/3_)O_3_]_(1−x)_ − [PbTiO_3_]_x_ (PMN-PT) is used^19–27^ due to its large piezoelectric charge constant^30^ *d*_33_ > 2000 pV m^−1^ and the fact that it is readily manufactured as single crystals. This, combined with the fact that the MPT in FeRh is highly sensitive to very small changes in lattice constant, suggests that the magnetoelectric coupling in this system can in principle be very large.

In practice, this coupling can be limited for several reasons. First, poor heteroepitaxy between the substrate and thin film leads to imperfect strain propagation, and may also induce intrinsic strains which act to diminish the effect of an external field^31^. Moreover, even in cases where perfect strain transmission from substrate to the first atomic layer of FeRh is a reasonable assumption, strain dissipates with distance from the interface as misfit dislocations develop^32,33^. Finally, a material under tensile strain in one direction is usually under compressive strain in the two orthogonal directions, and vice versa. This effect, quantified by Poisson’s ratio *ν*, can act to further quench magnetoelectric coupling in strain-mediated multiferroic systems^34^. For bulk FeRh *ν*_FeRh_ ≈ 0.31^35^, but this value may vary with film thickness and epitaxy^36^. Work to understand limitations to strain mediated magnetic manipulation in FeRh-based artificial multiferroics is therefore invaluable to the engineering of future devices based on this effect. This is especially true for PMN-PT, which has structural and dielectric phase transitions which can lead to a wide range of piezoelectric properties depending on composition, temperature and applied electric field^37^.

In this work, we demonstrate the presence of magnetoelectric coupling in a 21 nm thick FeRh thin film fabricated on an [001]-oriented PMN_28_-PT_72_ single crystal substrate, capped with a 1 nm layer of Pt to prevent oxidation. In order to quantify the magnetoelectric effects, we measure the magnetisation directly using a vibrating sample magnetometer which was modified to simultaneously apply electric and magnetic fields as a function of temperature. Through X-ray diffraction (XRD) measurements and variable-temperature vibrating-sample magnetometry (VSM) with in-situ applied electric fields, we show that the magnetoelectric coupling is quenched at temperatures above ∼ 100 °C, which can be attributed to a gradual structural phase transition in the PMN-PT. We demonstrate a large strain remanence effect in the PMN-PT substrate, which limits the magnetoelectric coupling on successive cycling of the applied electric field. Despite these limitations, we verify that through careful selection of the applied electric field range, it is possible to induce repeatable and deterministic all-electrical switching in this system over many electric field cycles.

## Results

**Figure 1:**
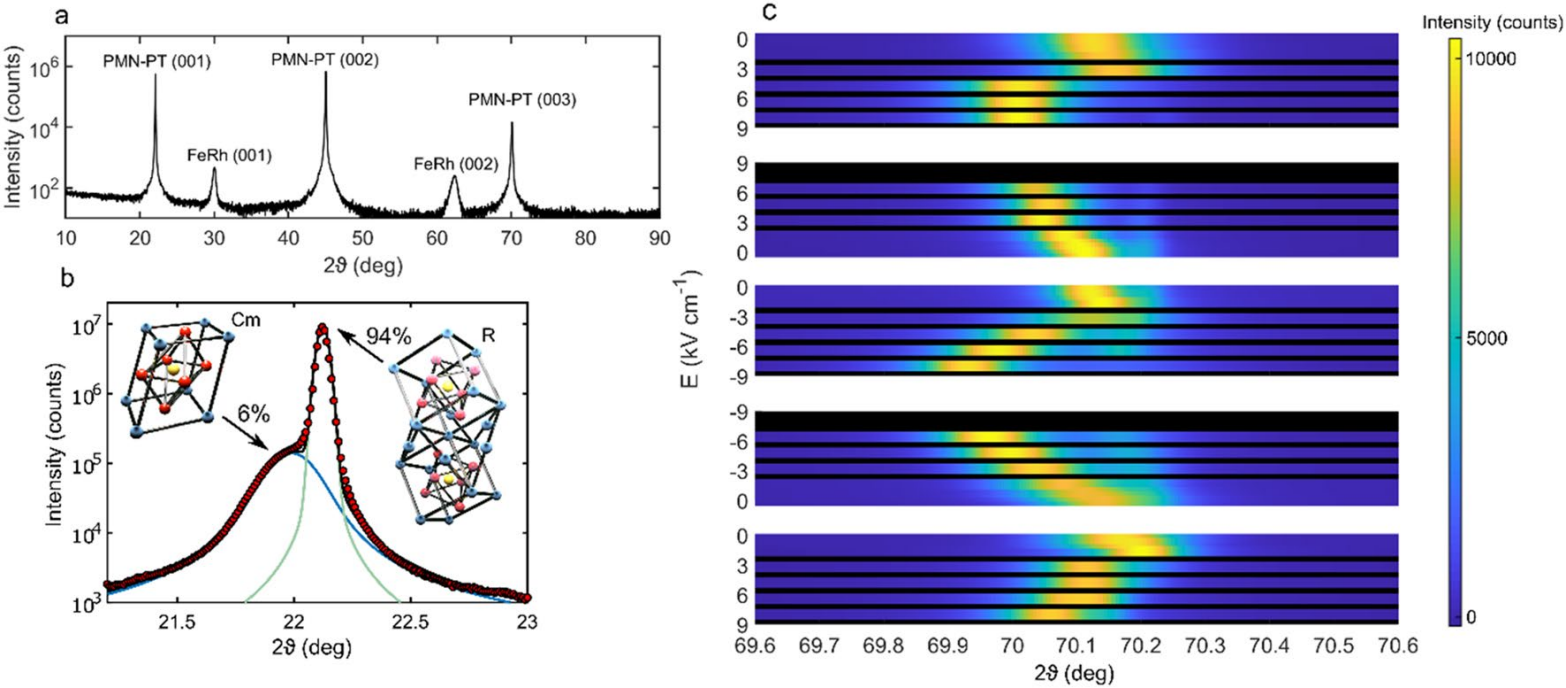
(**a**) XRD data for a ∼ 20 nm FeRh thin film fabricated onto an [001]-oriented PMN_28_-PT_72_ single crystal substrate. The presence of FeRh (001) and (002) diffraction peaks demonstrates the presence of B2-ordered FeRh. (**b**) XRD data taken about the PMN-PT (001) diffraction peak. A duel-peak diffraction is observed, with contributions from the rhombohedral and cubic monoclinic phases of PMN-PT. (**c**) XRD data about the PMN-PT (003) diffraction peak as a function of applied electric field *E*. The data show that increasingly positive or negative electric fields shift the diffraction peak towards lower ϑ, while changes in the applied field towards zero shift the peak towards larger ϑ.

The structural properties of the FeRh thin film were confirmed using X-ray reflectivity (XRR), as shown in Fig. S1 of the Supplementary Information. The roughness of the interface was determined to be 0.7 nm from modelling the XRR data. This compares favourably with FeRh grown on MgO using the same deposition process where the interfacial roughness was determined to be 1.5 nm^38^ and is significantly less than the value found for FeRh grown on FePt of 4.7 nm^39^. We therefore make the assumption that the interface is at least as good as for when FeRh is grown on these substrates, although determining the local structure of the interface would require a local probe such as transmission electron microscopy (TEM). The sample crystallinity was verified using X-ray diffraction (XRD), with all XRR/XRD measurements conduced in standard ϑ–2ϑ geometry, where 2ϑ is the angle of diffraction. The XRD data are shown in Fig. 1a. Here, the presence of FeRh (001) and (002) diffraction peaks at ∼ 30° and ∼ 62° respectively indicate the presence of FeRh in the B2 crystallographic phase. To test this quantitatively, Voigt profiles were fitted to each of these peaks (Fig. S2 of the Supplementary Information), which allows the crystallographic order parameter *S* to be calculated (details in the supplementary information). This analysis yields *S* = 0.81, which is comparable to values found for FeRh deposited on MgO elsewhere^31,40^, indicating a high degree of ordering in the FeRh thin film.

The XRD data also evidence a dual phase behaviour in the PMN-PT, with each diffraction peak having a shoulder at lower 2ϑ. To better understand this, XRD measurements were conducted on a blank substrate about the PMN-PT (001) diffraction peak with a step size of 0.01°. The resulting data were then fitted using a weighted combination of two Voigt profiles (see “Methods” section). The measured data with corresponding fits are shown in Fig. 1b. The temperature-composition phase diagram for PMN_x_-PT_1−x_ is not well understood^37^, with several different combinations of low symmetry phases reported to be present at *x* ∼ 0.3^41–43^, where there is a morphotropic phase boundary separating the relaxor ferroelectric rhombohedral phase of Pb(Mg_1/3_Nb_2/3_)O_3_ from the conventional ferroelectric tetragonal phase of PbTiO_3_. Given the compositional proximity of the substrates used here to the morphotropic phase boundary, and following the phase diagram constructed by Shuvaeva et al.^42^, it can be inferred that the largest phase fraction corresponds to the rhombohedral (R) phase, with the remainder of the substrate being in the monoclinic (Cm) phase. The fits to the data in Fig. 1b allow approximate phase fractions to be obtained from the ratio of peak areas. In order to account for the difference in structure factor for X-ray scattering, the NIST Inorganic Crystal Structure Database tool for simulating standardized powder X-ray diffraction patterns was used^44^, with input parameters from Slodczyk et al*.*^45^ and Singh et al.^46^ which describe PMN-PT in the rhombohedral and monoclinic phases respectively. This analysis indicates that the substrate is ~ 6% monoclinic and ~ 94% rhombohedral; the mixed phase composition likely results from stoichiometric inhomogeneity throughout the crystal.

To demonstrate the piezoelectric response of the substrate, XRD was also performed on a blank PMN-PT crystal with an in-situ electric field *E* applied along the [001] direction. The lattice plane spacing *d* is related to the diffraction angle by Bragg’s law: *nλ* = 2*d* sinϑ, where *n* = 3 is the order of diffraction and *λ* = 1.5406 Å is the wavelength of the incident X-rays. Thus, to measure the fine detail of the change in peak position Δϑ resulting from a change in lattice parameter Δ*d*, measurements were made over 2ϑ = (69.6 − 70.6)° with a resolution of 0.01°, i.e. spanning the PMN-PT (003) diffraction peak. The applied electric field was first cycled from (0 →  + 8 →  − 8 → 0) kV cm^−1^, then from (0 →  − 8 →  + 8 → − 8) kV cm^−1^ using smaller increments. The diffraction peaks from the latter cycle are shown in Fig. 1c. For the top electrode, a sputtered thin film (~ 10 nm) of Pt was used. It should be noted that the use of Pt in lieu of an FeRh thin film as the top electrode may subtly affect the quantitative features of the resulting piezoelectric response. The data show that the peak position undergoes a reversible change with the electric field, where increasingly positive or negative values of the field correspond to a positive Δ*d*, while changes in the field towards zero lead to a negative Δ*d*. To determine the value of the strain, the intensity versus 2ϑ data were fitted using weighted combinations of Voigt profiles for each value of the applied electric field, using data from both cycles (Supplementary Fig. S3). The data demonstrate that the rhombohedral and monoclinic phases exhibit independent piezoelectric behaviours. The field-induced perpendicular strain in the more prevalent rhombohedral phase ${\varepsilon }_{R}^{E}$(*E*) can be calculated using Bragg’s law, which leads to

1 $${\varepsilon }_{R}^{E}\left(E\right)= \frac{{d}^{E}-{d}^{E = 0}}{{d}^{E = 0}} = \frac{\text{csc} {\vartheta }_{R}^{E}-\text{csc} {\vartheta }_{R}^{E = 0}}{\text{csc} {\vartheta }_{R}^{E=0}}.$$

**Figure 2:**
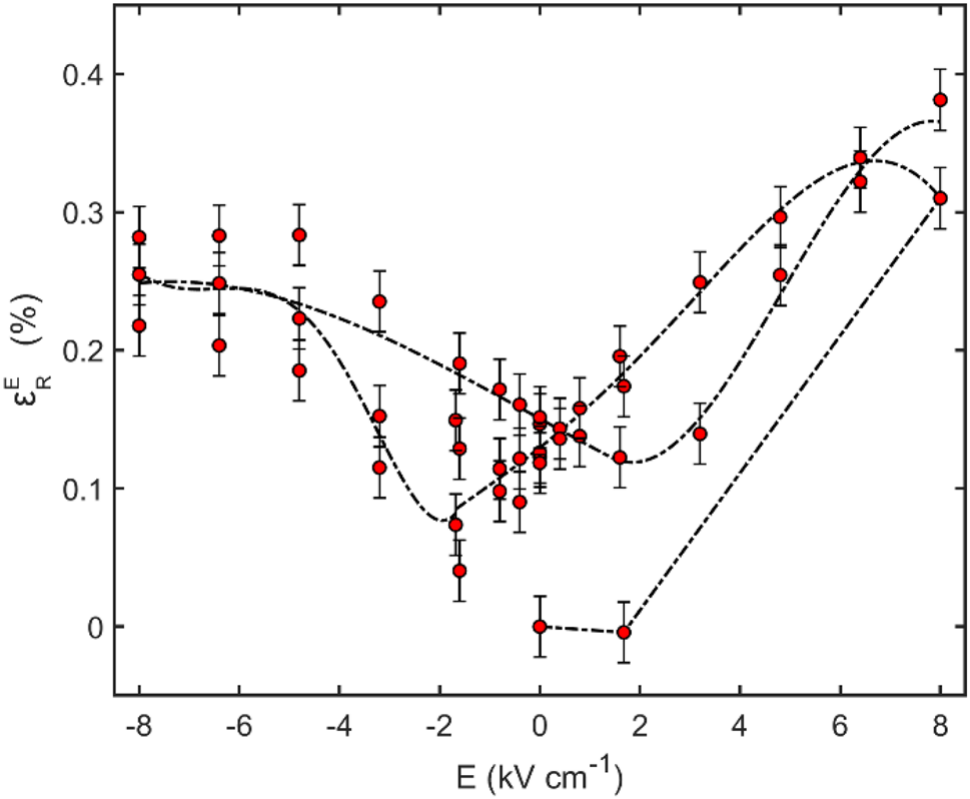
The perpendicular strain ${\varepsilon }_{R}^{E}$(*E*) in the rhombohedral phase of PMN-PT is plotted against applied electric field *E*. The data demonstrate a large, butterfly-like piezoelectric response, with the strain reaching a maximum of 0.38% at *E* = 8 kV cm^−1^. The dashed curve is a guide-to-the-eye constructed by stitching polynomial fits to the data.

Using Eq. (1) and the data of Supplementary Fig. S3, the strain in the rhombohedral phase with an electric field applied along the [001] direction is given in Fig. 2. The data show that on first applying + 1.68 kV cm^−1^ to the virgin state, there is very little change to the lattice parameter. Increasing the field to + 8 kV cm^−1^ leads to a significant change, with the strain reaching ∼ 0.3%. By subsequently cycling the field between + 8 kV cm^−1^ and − 8 kV cm^−1^, the piezoelectric response traces out a butterfly curve, where both highly positive and highly negative values of the applied electric field produce a large positive strain.

**Figure 3:**
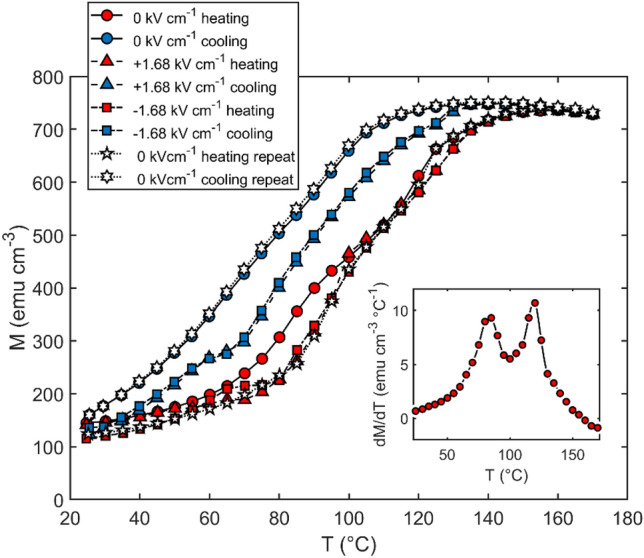
Thermomagnetic measurements of PMN-PT/FeRh with constant applied electric fields of 0 kV cm^−1^ (circles), + 1.68 kV cm^−1^ (triangles), and − 1.68 kV cm^−1^ (squares). Also shown are measurements made after resetting the electric field to zero (pentagrams and hexagrams for heating and cooling respectively). In the inset, d*M*/d*T* is shown for the heating branch of the zero electric field measurement.

To demonstrate the effect of the piezoelectric response identified in Fig. 2 on the magnetic properties of the FeRh, magnetisation loops were measured as a function of sample temperature at constant electric field, using VSM with in-situ applied voltages. Prior to measurement, a + 8 kV cm^−1^ poling field was applied to the sample. Magnetisation curves were then measured over a temperature range *T* = (25–165) °C, using *E* = 0 kV cm^−1^ and *E* =  ± 1.68 kV cm^−1^, with an applied magnetic field *H* = 1 kOe for all measurements. The data are shown in Fig. 3. In the zero electric field case, on heating to 175 °C (red circles) the film reaches a maximum magnetisation of 750 emu cm^−3^, consistent with previous observations from FeRh films of similar thickness^47,48^. The cooling curve (blue circles) reveals a hysteresis of ∼ 25 °C, which is also typical for FeRh of this thickness^49^.

Measurements with *E* =  + 1.68 kV cm^−1^ (triangles) and *E* =  − 1.68 kV cm^−1^ (squares) applied to the sample demonstrate that in both cases FM phase nucleation is suppressed by the electric field on heating up to *T* ∼ 100 °C, as evidenced by the reduced magnetisation compared to the initial 0 kV cm^−1^ case over this temperature range. This magnetoelectric effect is attributed to piezoelectric strain coupling across the film/substrate interface^20,24,25^. The fact that the effect of suppressing development of the FM phase is the same for positive and negative electric fields indicates that ± 1.68 kV cm^−1^ is beyond the ferroelectric coercivity of the PMN-PT at the onset temperature of the MPT.

At *T* ∼ 100 °C, the 0 kV cm^−1^ and ± 1.68 kV cm^−1^ heating curves begin to coincide, indicating that there is no magnetoelectric effect at these temperatures. The Curie temperature *T*_C_ for PMN-PT is ∼ 150 °C^50^; thus, above this temperature no magnetoelectric coupling is expected, but this does not account for the observed convergence of zero-electric-field and applied electric- field data at T ∼ 100 °C. This effect is explored further in the “Discussion” section. The cooling curves demonstrate a reduced magnetisation compared to the zero-electric field case for all temperatures below 140 °C, further demonstrating the piezoelectric strain-mediated stabilisation of the AF phase.

Interestingly, after cooling under − 1.68 kV cm^−1^ and resetting the applied electric field to 0 kV cm^−1^, the suppression of the FM phase persists on heating, with the resulting magnetisation curve (pentagrams) following the − 1.68 kV cm^−1^ case. This remanent state disappears on heating beyond *T*_C_ of the PMN-PT, hence the subsequent cooling curve (hexagrams) coincides with the initial zero-electric field case.

**Figure 4:**
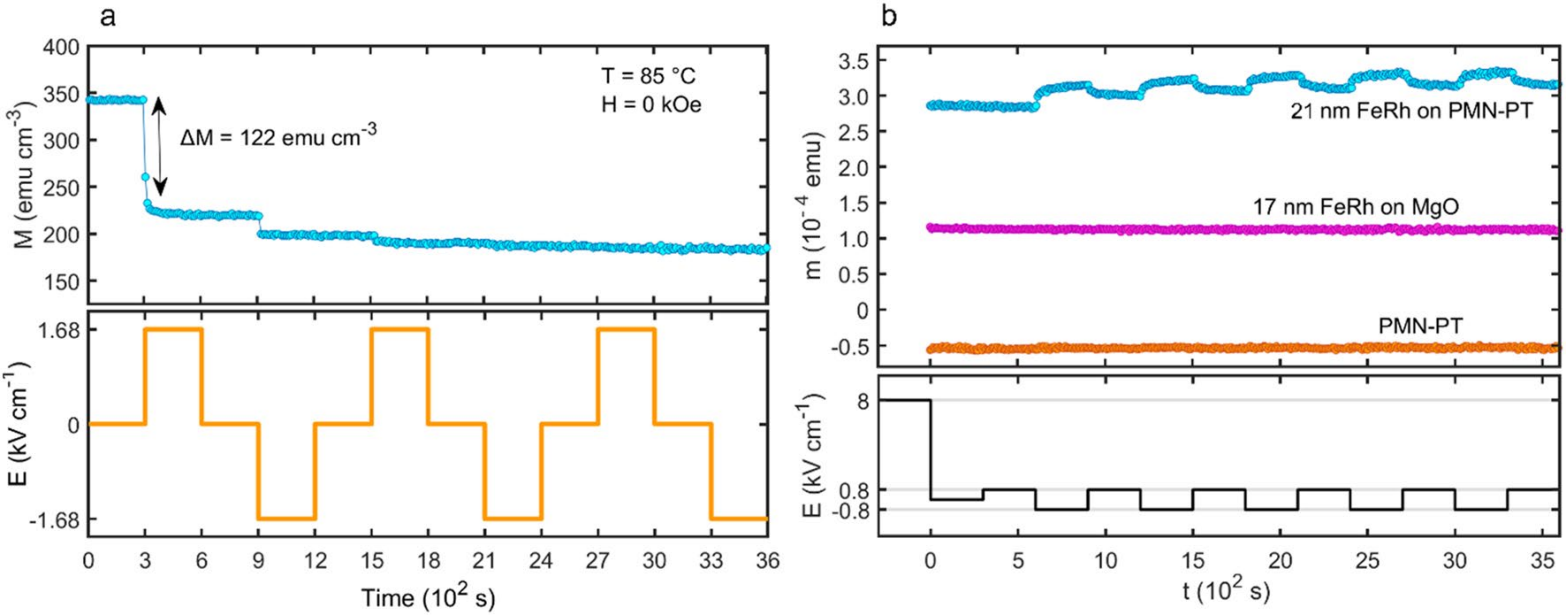
(**a**) The magnetisation of PMN-PT/FeRh as the applied electric field is switched between + 1.68 kV cm^−1^ and − 1.68 kV cm^−1^. On first increasing the electric field, the magnetisation is significantly reduced by the strain mediated magnetoelectric effect. Subsequent changes to the field produce diminishingly small changes to the magnetisation due to the strain being in a highly remanent state. (**b**) Repeatable switching in PMN-PT is demonstrated by applying an alternating ± 0.8 kV cm^−1^ electric field after poling at + 8 kV cm^−1^. Measurements were conducted at 85 °C.

To explore the potential for all-electrical switching, the in-plane magnetisation was measured as a function of varying electric field at constant temperature and zero applied magnetic field. These measurements were made at 85 °C, which was chosen to coincide with the first peak in d*M*/d*T* on the zero-electric field heating branch (inset to Fig. 3). The sample was first cooled from 200 to 25 °C in a zero magnetic field and no electric field, then heated to 85 °C under 1 kOe magnetic field and zero electric field, such that the strain state is reset but regions of FM ordering are preferentially aligned. The magnetic field was then set to 0 kOe, and electric field was repeatedly cycled from 0 kV cm^−1^ →  + 1.68 kV cm^−1^ →  − 1.68 kV cm^−1^ → 0 kV cm^−1^, remaining at each value for 300 s. This cycle of the applied electric field and resulting variation in *M* are shown in Fig. 4a. The data show that the strain remanence effect leads to an irreversible reduction in the FeRh magnetisation on first application of the electric field. However, on subsequent cycles the effect reduced asymptotically.

To reduce the effect of remanent strain during electric field cycling, the isothermal switching experiment was repeated but with *E* switched between ± 0.8 kV cm^−1^ after application and removal of a + 8 kV cm^−1^ poling field. The motivation for this was as follows: after applying the large poling field, the PMN-PT is in a high remanence state. By subsequently switching between values of the electric field which are lower than the ferroelectric coercive field, the strain produced is approximately linear in the electric field (see Fig. 2), allowing a large range of strain states to be generated. A similar approach to achieving magnetoelectric effects in PMN-PT/La_2/3_Ba_1/3_MnO_3_ was reported by Zhou et al.^51^, where the application of minor electric field loops allowed magnetoelectric switching to be achieved. The resulting in-plane magnetic moment *m* as a function of time is shown in Fig. 4b, which also shows the same measurements from a 17 nm FeRh film on MgO and a blank PMN-PT substrate for comparison.

## Discussion

The strain vs. electric field data shown in Fig. 2 reveal that the PMN-PT strain state does not return to zero at any point in the sequence of applied electric fields. This indicates that an irreversible lattice deformation has been induced on application of the initial poling field. For a prototypical ferroelectric it is expected that as an electric field is applied along an axis antiparallel to the initial poling direction, the strain should pass through zero and become negative until the poling field is reached in the opposite direction, at which point the strain increases again. This behaviour is not observed in the data shown in Fig. 2. The remanent strain seen here effectively limits the useful range of strain values which can be accessed to actuate the FeRh lattice and affect a change to its magnetisation.

Despite this strain-remanence effect, the constant-electric-field thermomagnetic data of Fig. 3 demonstrates that a notable magnetoelectric coupling is present in this system. The maximum magnetoelectric effect is observed on the cooling curve at 70 °C, where the magnetisation with + 1.68 kV cm^−1^ electric field is 297 emu cm^−3^, i.e. 138 emu cm^−3^ lower than in the zero-field case. This corresponds to a magnetoelectric coupling coefficient of *α* = 670 (equivalent to 1.03 × 10^−6^ s m^−1^ in SI units) where *α* is defined by *α* = 4π(∂*M*/∂*E*). This maximum coupling strength is greater than those observed by Fina et al.^24^ (*α* ≈ 185, or 6 × 10^−7^ s m^−1^, where no magnetic field was applied during measurement) and Hu et al.^21^ (*α* ≈ 61.7, or 2 × 10^−7^ s m^−1^), but is notably smaller than that reported by Cherifi et al. for FeRh on BaTiO_3_^15^ (*α* ≈ 4937, or 1.6 × 10^−5^ s m^−1^). The large variation in PMN-PT/FeRh magnetoelectric coupling in across the literature reflects the sensitivity of the piezoelectric response to the compositional, structural, and dielectric properties of PMN-PT. Indeed, a similar analysis to that presented in Fig. 1b yields appreciably different compositional phase fractions for PMN_28_-PT_72_ supplied from three different manufacturers (Supplementary Fig. S4), with the monoclinic fraction ranging from 6 to 25% across the three suppliers tested.

The initial zero-electric field thermomagnetic measurement (red and blue circles in Fig. 3 for heating and cooling respectively) shows that the FeRh has a substantial magnetisation of 141 emu cm^−3^ at room temperature. By contrast, for FeRh on MgO the magnetisation at room temperature is usually very close to zero^47^. This residual FM component may result from poor epitaxy at the PMN-PT interface and/or inadvertent doping with highly mobile Pb atoms^52^. For either case, the effect would be localised at the interface, where the strain coupling is expected to be strongest. This reduces the magnitude of the change in magnetisation that can be induced as a result of magnetoelectric coupling. Reducing this residual magnetization is one of the outstanding challenges in enhancing the effect of electric fields on the magnetic state of multiferroic PMN-PT/FeRh.

In has been reported that on heating from room temperature, PMN-PT undergoes a change in crystal structure from rhombohedral to tetragonal symmetry, beginning at *T* ≳ 100 °C^42,50,53^. The fact that the 0 kV cm^−1^ and ± 1.68 kV cm^−1^ heating curves of Fig. 3 begin to converge at T ∼ 100 °C suggests that there is a significant strain effect associated with this structural phase transition, whereby even in the zero-electric field case development of the FM phase is inhibited. This further evidenced by the form of d*M*/d*T* for the heating branch of the zero-electric field measurement, shown in the inset to Fig. 3. The local minimum at *T* ∼ 100 °C results from the strain due to the structural phase transition, which favours the AF phase and thus suppresses the MPT from AF to FM ordering. This structural phase instability in PMN-PT, which has been overlooked in the literature concerning PMN-PT/FeRh multiferroics thus far, has a clear effect to limit the magnetoelectric coupling to an adjoining FeRh thin film at a temperature range close to that expected for device operation.

When the electric field is cycled between ± 1.68 kV cm^−1^ (Fig. S4 of the Supplementary Information), the magnetisation is initially reduced by Δ*M* = 122 emu cm^−3^, which corresponds to a magnetoelectric coupling coefficient of *α* = 592 (9.15 × 10^−7^ s m^−1^), i.e. a quantitatively similar coupling strength to that observed in constant-electric-field thermomagnetic measurements under *E* =  ± 1.68 kV cm^−1^ and *H* = 1 kOe. Consistent with the observation that the strain remains in a state of high remanence when the electric field is removed (Fig. 2), subsequent changes in *E* do not lead to a comparably large magnetic modulation. Rather, each time *E* is driven away from zero, *M* reduces by an asymptotically diminishing amount, indicating that successive straining of the substrate beyond remanence has a limited effect in stabilising the AF phase. In contrast, on successive cycling of the electric field between ± 0.8 kV cm^−1^, the magnetic moment is controllably switched, with an average change Δ*m* = 1.5 × 10^−5^ emu, providing a magnetic modulation of ∼ 5%. Repeatable electric field switching over many cycles in PMN-PT/FeRh has also been reported by Clarkson et al*.*^54^ In their study, the rotation of the magnetic anisotropy axis in FeRh where both AF and FM phases were present was demonstrated using electrical transport measurements. The all-electrical switching behaviour which we observe for PMN-PT/FeRh is not observed in the MgO/FeRh or blank PMN-PT (Fig. 4b), verifying that it is due to magnetoelectric coupling of the FeRh to the piezoelectric substrate. The magnetic moment of the MgO/FeRh is substantially lower than that of the PMN-PT/FeRh (even when the differences in film thickness are considered), which reflects the reduced MPT temperature of the sample fabricated on PMN-PT.

## Conclusions

In this work, we identify key features of the temperature-dependent magnetoelectric coupling in a PMN-PT/FeRh artificial multiferroic. The data demonstrate that suitable values of the electric field may be chosen that produces a repeatable, deterministic, and all-electrical switching in PMN-PT/FeRh. We show that the ferroelectric rhombohedral to ferroelectric tetragonal phase transition which occurs in PMN-PT at ∼ 100 °C plays a significant role to inhibit development of the FM phase on heating, even in the case of zero electric field, an important consideration for effective exploitation the magnetoelectric coupling in functional devices. It has also been shown that the PMN-PT piezoelectric response can exhibit a strain remanence effect, whereby over the range of electric fields applied in this experiment (− 8 kV cm^−1^ to + 8 kV cm^−1^), the strain in the PMN-PT does not revert to the initial zero-electric field state. Hence, there is an irreversible component of the strain which is generated on first application of the electric field but which cannot be removed by reversing the electric field alone. Thus, the range of strain values which can be accessed by cycling the applied electric field is limited by this effect.

## Methods

### Sample growth

The sample was fabricated via dc magnetron sputtering from a stoichiometric Fe_50_Rh_50_ target onto a PMN-PT single crystal substrate using an Ar^+^ working gas. One side of the substrate was coated with Cr/Au, which acted as a bottom electrical contact. On the other side, an FeRh flash layer (∼ 3 nm) was first deposited at room temperature using 100 W power and 3 mTorr working pressure in order to provide a good surface on which to grow the remainder of the film. This layer was subsequently annealed at 600 °C for two hours, after which the remaining 18 nm was deposited at 600 °C using 100 W dc power and 3 mTorr Ar^+^ working pressure. The sample was left to cool in the vacuum chamber for four hours before a cold Pt capping layer was deposited in order to avoid oxidation of the FeRh thin film and to provide a top electrical contact. The base pressure of the system prior to deposition was below 5 × 10^−8^ Torr.

### X-ray measurements

All XRR and XRD data were conducted in standard ϑ–2ϑ geometry using a Rigaku SmartLab system equipped with a two-bounce Ge(220) monochromator. This apparatus produces X-rays at the CuK*α* edge, i.e. with a wavelength of *λ* = 1.5406 Å. To extract layer thickness, densities, and roughnesses from the XRR measurements, dynamical simulations were fitted to the data using the freeware GenX^55^. Details of the fitting procedure are provided in the supplementary information. For the full spectrum XRD profile, a step size of 0.02° was used. This was reduced to 0.01° for the analysis of PMN-PT phase composition. XRD measurements with in-situ applied electric fields were conducted using a purpose-built sample stage and a Keithley 2450 source-meter. Changes to the electric field were applied incrementally so as to avoid overstressing the PMN-PT and to monitor for electrical shorting. For the order parameter analysis of FeRh, Voigt profiles were fitted to (001) and (002) peaks using the using the Levenberg Marquardt iteration algorithm. Fitting parameters included the peak centres, baseline, Lorentzian and Gaussian widths, and peak areas. For analysis of XRD data from PMN-PT, each peak was fitted using a weighted combination of two neighbouring Voigt profiles, with the weighting factor being an additional fitting parameter.

### Magnetometry

All magnetometry was conducted using a MicroSense Model 10 vector vibrating sample magnetometer (VSM), which allows the sample temperature to be varied. Background subtraction was achieved by subtracting the thermomagnetic response of a blank PMN-PT substrate. Measurements with in-situ applied electric fields were achieved via a modification to the sample mounting environment to accommodate the application of high voltages produced by a Keithley 2450 source-meter.

## Supplementary Information

Supplementary Information.

## Data Availability

The data that support the findings of this study are available from the corresponding author upon request.
