# Supplementary material for: Repeatable and deterministic all electrical switching in a mixed phase artificial multiferroic
Source: Sci Rep. 2022 Mar 29;12:5332. doi: 10.1038/s41598-022-09417-0 (PMC8964689; doi:10.1038/s41598-022-09417-0)
Supplement: Supplementary file 1 — Supplementary Information. [file 41598_2022_9417_MOESM1_ESM.docx]

Repeatable & deterministic all electrical switching in a mixed phase artificial multiferroic – supplementary information

W. Griggs^1,*^ and T. Thomson^1^

^1^NEST Research Group, The Department of Computer Science, The University of Manchester, Oxford Road, Manchester, M13 9PL, United Kingdom

____________________________________

*William.griggs@manchester.ac.uk

FeRh/PMN-PT structural analysis

The thickness, density, and roughness of each layer in the PMN-PT/FeRh/Pt sample were probed using X-ray reflectivity (XRR) in standard ϑ - 2ϑ geometry, as shown by the blue data in Fig. S1(a). A dynamical simulation of the XRR profile (solid red line) was fitted to the data using the GenX reflectivity package [S1], allowing the scattering length density (SLD) profile to be extracted (Fig. S1(b)). The fit was achieved using a logarithmic figure of merit (FOM) as the more usual reduced 𝜒^2^ FOM is inappropriate due to the fact that systematic uncertainties (for example arising due to an imperfect Gaussian beam footprint) tend to dominate XRR measurements. The functional form of this FOM is given by

$$\mathrm{FOM}_{\log}= \frac{1}{N-p}\sum_{i} \log_{10} y_{i}-\log_{10} s_{i}$$

where *y*_i_ and s_i_ are the i^th^ measured and simulated data points respectively, *N* is the number of data points, and *p* is the number of fitted parameters.


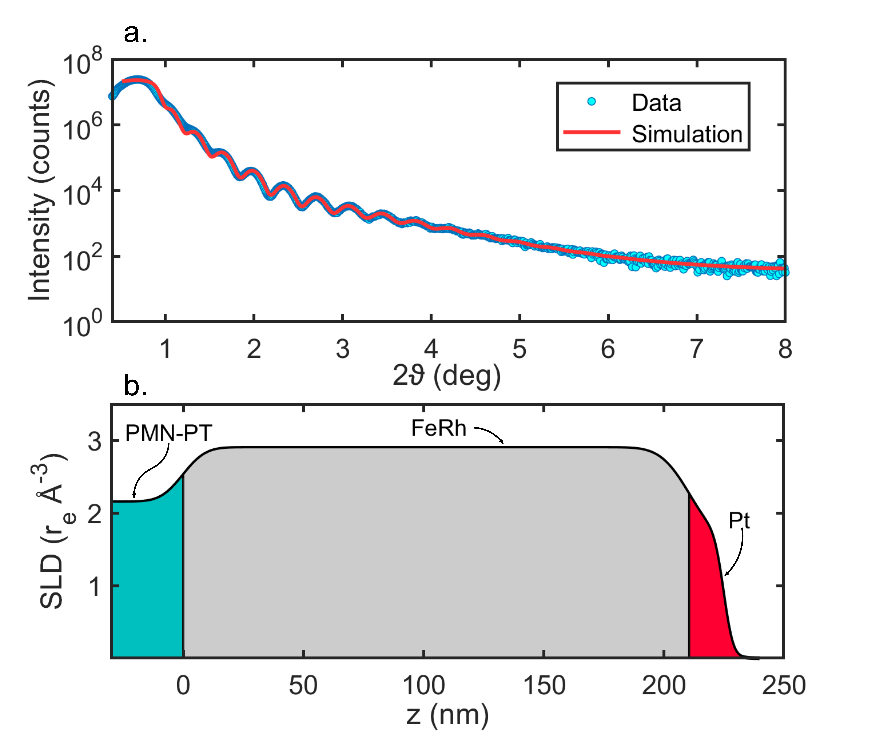


Figure S1: **(a)** XRR data (blue circles) from the PMN-PT/FeRh/Pt sample, with corresponding fitted simulation (red line). **(b)** The resulting SLD profile.

The fitted XRR parameters are provided in Table S1.

| Layer | Thickness (nm) | Density (f.u./Å^3^) | Roughness (nm) | FOM_log_ |
| --- | --- | --- | --- | --- |
| Pt | 1.4 ± 0.2 | 0.022 ± 0.009 | 1.4 ± 0.2 | 4.47 × 10^-2^ |
| FeRh | 21.1 ± 0.4 | 0.041 ± 0.001 | 0.9 ± 0.2 |  |
| PMN-PT | - | 0.0164 ± 0.001 | 0.7 ± 0.2 |  |

Table S1. Thicknesses, densities, and roughnesses as obtained from the fitted XRR data. Uncertainties were calculated via a Bayesian approach using the Bumps uncertainty modelling package [S2].

To verify the crystallinity of FeRh deposited on PMN-PT, Voigt profiles were fitted to FeRh (001) and (002) peaks measured using X-ray diffraction (XRD) in a standard 𝜗-2𝜗 geometry. These fits and data are shown in Fig. S2.


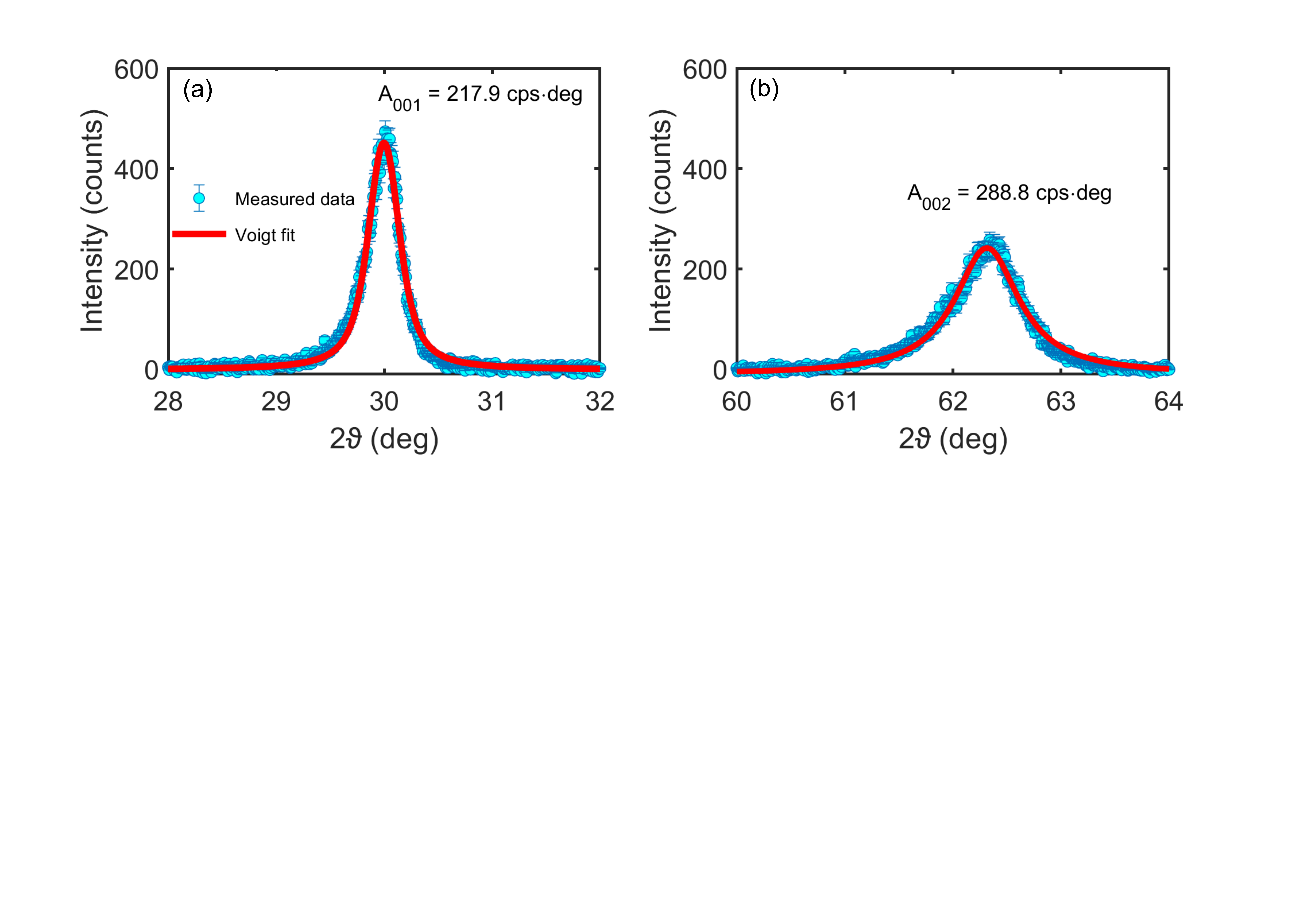


Figure S2: Fits (solid red lines) to **a.** FeRh (001) and **b.** FeRh (002) diffraction peaks acquired through XRD (blue data) in 𝜗-2𝜗 geometry, where 2𝜗 is the diffraction angle. The areas under each peak A_001_ and A_002_ are also provided.

For each diffraction peak, the fit comprises a single Voigt profile with variable peak centre, Lorentzian width, Gaussian width, and integrated intensity. The order parameter *S*, which quantifies the degree of crystallographic order where *S* = 0 represents a random distribution of scattering species and *S* = 1 is a perfect crystal, can be calculated from the areas underneath the (001) and (002) peaks, denoted A_001_ and A_002_ respectively, using [S3]

|  | $S= \frac{1}{1.07}\sqrt{\frac{A_{001}}{A_{002}}} .$ | (S1) |
| --- | --- | --- |

This analysis yields *S* = 0.81 for the FeRh thin film studied in this investigation, indicating good crystallographic quality and consistent with the value of *S* = 0.82 found in our previous work on MgO(001)/FeRh thin films [S4].

Piezoelectric characterisation of PMN-PT

To characterise the piezoelectric response of the PMN-PT substrate, XRD data were taken about the PMN-PT (003) peak for a series of values of the applied electric field *E*. These data are shown in Fig. S3. Also shown are fits to the data, which comprise weighted sums of two Voigt profiles in order to capture diffraction peaks from the monoclinic and rhombohedral phases. Fitted parameters include peak centres, Gaussian widths, Lorentzian widths, and integrated intensities. In calculating values of the induced strain, the peak centres are the critical parameter; therefore, in cases where the two peaks overlapped no optimisation for peak widths or areas was performed. The order of the measurements is indicated by the indices in the top left of each panel.


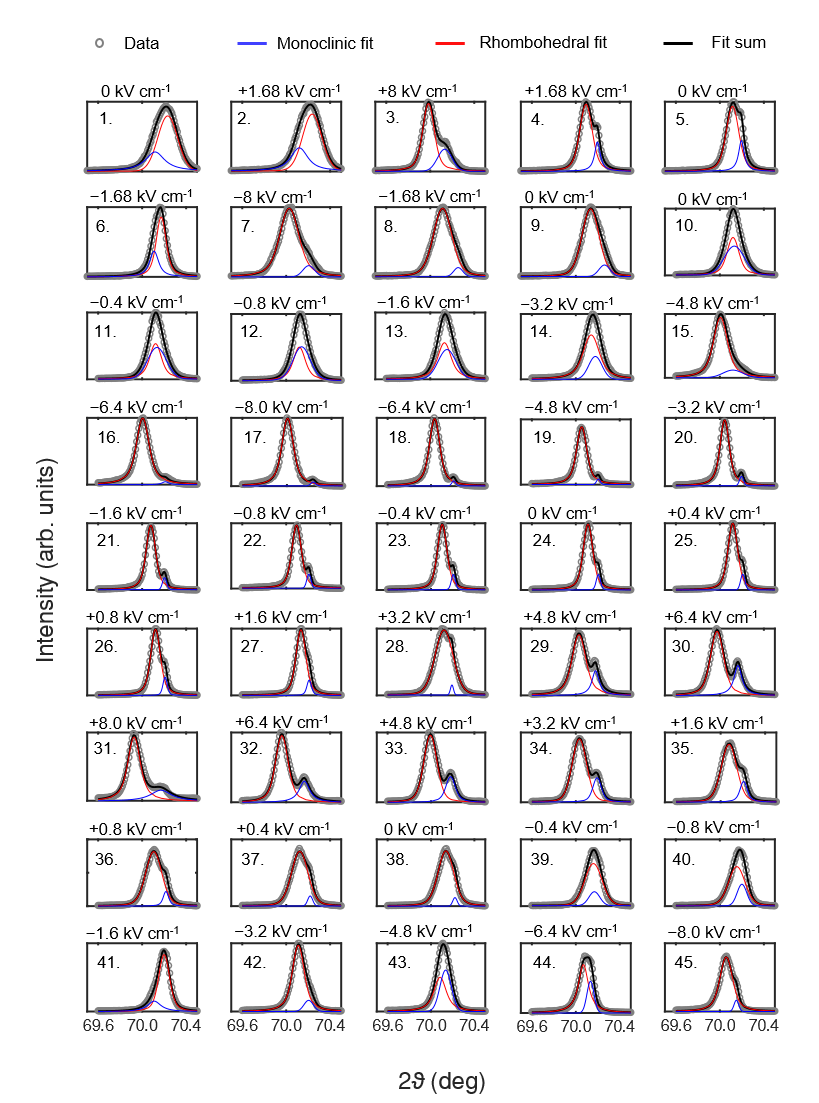


Figure S3: Fits to the PMN-PT (003) XRD peak over a series of applied electric fields. Each fit comprises a sum of two Voigt profiles, which result from the monoclinic and rhombohedral phases. The order of measurement is indicated by the numbers in the top left of each plot.

Comparison of PMN-PT suppliers

A comparison of the structural phase composition of PMN-PT from three separate suppliers was conducted using XRD. Quantitative abundances of structural phase fractions were obtained by fitting Voigt profiles to (001) diffraction peaks, which in all cases provide evidence for two phases being present. Due to the stoichiometric proximity to the morphotropic phase boundary in PMN-PT, it is assumed that the most prominent peak in each case corresponds to the rhombohedral phase (R), with the other peak resulting from the cubic monoclinic phase (Cm). Each diffraction profile was first corrected for the background signal by subtracting a fit to the baseline, after which a sum of two Voigt profiles was fitted to the peak. Fitted parameters include the individual peak centres, Gaussian and Lorentzian widths, peak areas, and a weighting parameter. The measured data (red circles) and corresponding fitted Voigt profiles (black lines) are shown in Fig. S4. Also shown are the individual contributions from rhombohedral (green curves) and monoclinic (blue curves) phases. The data show that the substrates each comprise a different composition of the two structural phases, and, from Fig. S3, it is evident that these phases have different piezoelectric responses. Therefore, the magnetoelectric coupling in a PMN-PT/FeRh artificial multiferroic structure is expected to vary significantly with substrate supplier.


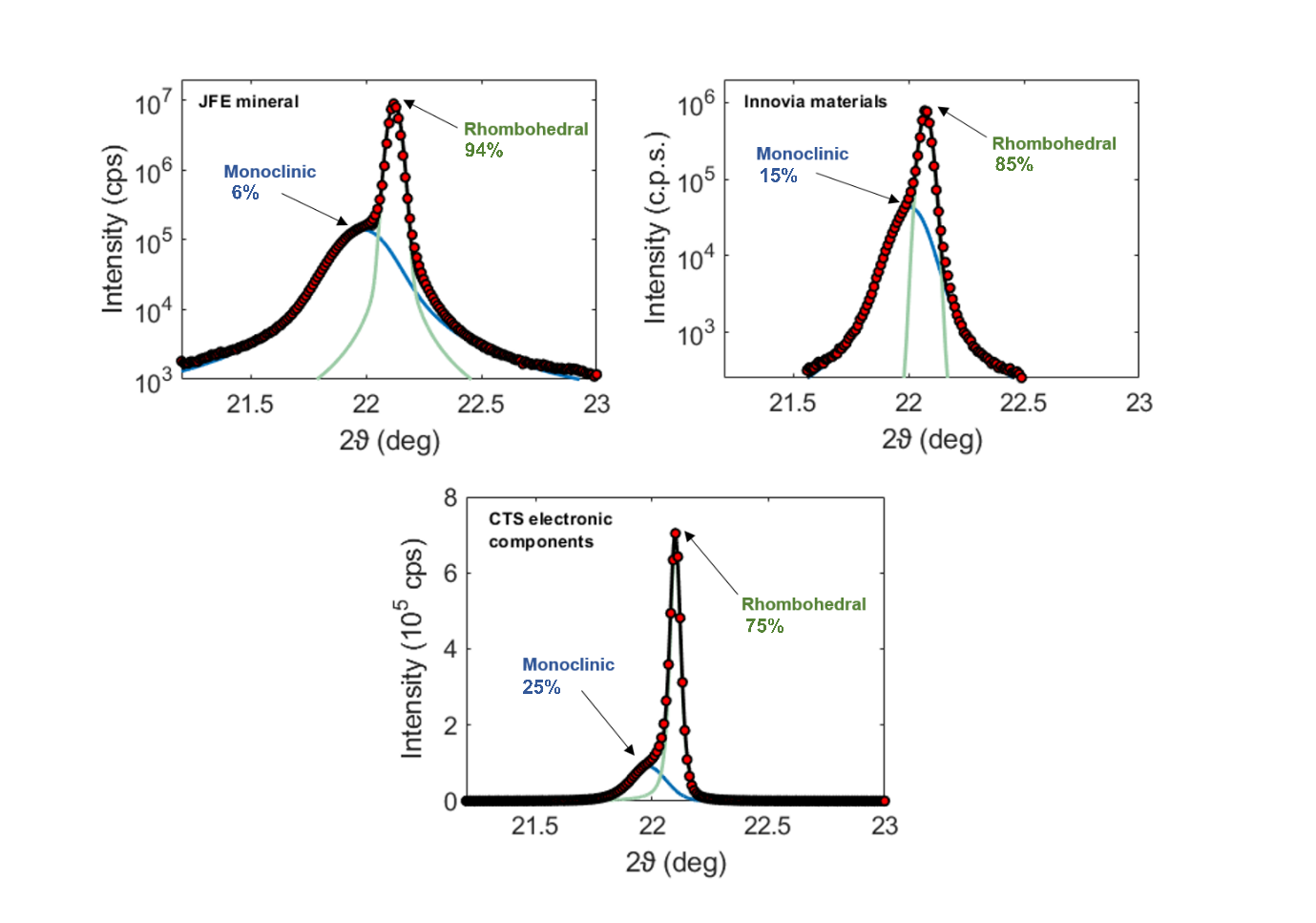


Figure S4: The (001) diffraction peaks from PMN-PT as supplied by three different manufacturers. In all cases a shouldered peak is present, evidencing a the coexistence of rhombohedral and monoclinic phases. Fits to the data comprising weighted sums of two Voigt profiles are also shown (black lines), alongside individual contributions corresponding to the monoclinic (blue lines) and rhombohedral (green lines) phases.

Supplementary references

| [S1] | M. Björck and G. Andersson, GenX: an extensible X-ray reflectivity refinement program utilizing differential evolution, J. Appl. Cryst., vol. 40, p. 1174–1178 (2007). |
| --- | --- |
| [S2] | P. A. Kienzle et al., Bumps (Version 0.8.0) [Computer Software] (2011), Retrieved November 2nd, 2021. |
| [S3] | C. L. Graët, Sputter Growth and Characterization of Metamagnetic B2-ordered FeRh Epilayers, J. Vis. Exp., vol. 80, no. e50603 (2013). |
| [S4] | W. Griggs et al., Depth selective magnetic phase coexistence in FeRh thin films, APL Mater., vol. 8, no. 121103 (2020). |
